# SRPX2 exhibits neuroprotective effects in neural stem cells: inhibition of OGD/R-stimulated apoptosis and oxidative stress

**DOI:** 10.1515/biol-2025-1182

**Published:** 2026-01-13

**Authors:** Pan Li, Jie Hua

**Affiliations:** Department of Pediatrics, Hangzhou Ninth Hospital, Qiantang District, Hangzhou City, Zhejiang Province, 311225, China

**Keywords:** hypoxic-ischemic encephalopathy (HIE), sushi repeat-containing protein X-linked 2 (SRPX2), apoptosis, oxidative stress, wnt/β-catenin pathway

## Abstract

Hypoxic-ischemic encephalopathy (HIE) is a major cause of infant morbidity as well as mortality. Neural stem cells (NSCs) is essential for brain development and function, and the role of NSCs in HIE is crucial and deserves further study. Sushi repeat-containing protein X-linked 2 (SRPX2) is a novel chondroitin sulfate proteoglycan which has multiple biological functions, such as cell growth and adhesion. However, SRPX2 is rarely reported in HIE process, and the mechanism is unclear. Herein, we aimed to uncover the role of SRPX2 in the progression of HIE. The oxygen-glucose deprivation/reoxygenation (OGD/R) model was established as a HIE cell model. We revealed that SRPX2 improves survival of OGD/R-stimulated NSCs. SRPX2 inhibited apoptosis of OGD/R-stimulated NSCs. Further data confirmed SRPX2 restrained oxidative stress of OGD/R-stimulated NSCs. Mechanically, SRPX2 activated the Wnt/beta-catenin pathway, and therefore suppressed the apoptosis as well as oxidative stress of OGD/R-stimulated NSCs. Collectively, SRPX2 suppressed apoptosis as well as oxidative stress of OGD/R-stimulated NSCs by activating Wnt/β-Catenin pathway.

## 1 Introduction

Hypoxic-ischemic encephalopathy (HIE) is a major cause of infant morbidity as well as mortality, affecting about 60 % of preterm infants [1]. HIE can cause severe brain damage, which can lead to the development of neurological disorders [1], 2]. Current clinical treatment plans include hypothermia, anticonvulsants, and electrolyte treatment, and treatment with medications such as atropine as well as epinephrine [3], 4]. There is still a lack of understanding of HIE pathophysiology as well as the mechanisms involved, so there is a need to investigate alternative strategies to replace or expand current treatment options. The precise control of self-renewal and differentiation of neural stem cells (NSCs) is essential for brain development and function [5], [6], [7]. In addition, Oxygen-Glucose Deprivation/Reoxygenation (OGD/R) could induce HIE [8]. Their destruction can lead to serious birth defects and life disorders. Therefore, the role of NSCs in HIE is crucial and deserves further study.

Sushi repeat-containing protein X-linked 2 (SRPX2) is a novel chondroitin sulfate proteoglycan, which plays a critical role in cell growth, adhesion, etc. [9]. SRPX2 can increase the proliferation as well as motility of sarcoosteoma cells and improve the prognosis of patients by regulating the Hippo signaling pathway [10]. SRPX2 increases proliferation and inhibits apoptosis of colon cancer cells [11]. SRPX2 can also promote the malignant process of pancreatic cancer, non-small cell lung cancer, esophageal cancer [12], [13], [14], etc. Notably, SRPX2 promotes angiogenesis and reduces infarct volume in a rat middle cerebral artery occlusion (MCAO) model, with knockdown exacerbating blood-brain barrier disruption – highlighting its endogenous neuroprotective role in ischemic injury [15]. However, animal studies have found that traumatic brain injury (TBI) in rats induces a decrease in SRPX2 expression and a decrease in the number of SRPX2 immune response neurons in the bilateral hypothalamus [16]. Furthermore, recent evidence suggests that SRPX2 may contribute to tissue remodeling and angiogenesis, processes critical to neurological recovery following ischemic injury. Thus, exploring its role in neuroprotective strategies could offer novel therapeutic insights for managing HIE.

This study was aimed to uncover the role of SRPX2 in HIE in a cell model and explore the mechanism. We revealed that SRPX2 suppressed OGD/R-stimulated apoptosis and oxidative stress, therefore suppressing HIE progression.

## 2 Materials and methods

### 2.1 Cell culture, transfection and treatment

Neural stem cells (NSCs) were purchased from the Chinese Academy of Sciences (Catalog No. CB-ACL-0098) and characterized by their ability to proliferate and differentiate into neuronal and glial lineages as indicated by the supplier’s specifications. The commercially obtained neural stem cells were sourced in compliance with the cell bank’s ethical protocols. Cells were cultured in Dulbecco’s Modified Eagle Medium (DMEM) supplemented with B27, EGF (20 ng/mL), and bFGF (20 ng/mL). The transfection of NSCs with pcDNA3.1-SRPX2 plasmids was transiently performed using Lipofectamine 3,000 reagent (Thermo Fisher Scientific, USA) according to the manufacturer’s instructions. For OGD, cells were incubated in glucose-free DMEM (ThermoFisher #11966025) supplemented with 0 % FBS within a hypoxia chamber (Stemcell Technologies) flushed with 1 % O_2_/94 % *N*_2_/5 % CO_2_ (O_2_ level verified by OX-500 probe), followed by reoxygenation in complete DMEM (4.5 g/L glucose + 10 % FBS) under standard conditions (5 % CO_2_/95 % air). Cells were transfected with pcDNA3.1-SRPX2 plasmids or negative control plasmids 24 h before initiating OGD/R treatment. Cells were transiently transfected using Lipofectamine 3,000 (Thermo Fisher #L3000015) with 2 μg plasmid DNA (pcDNA3.1-SRPX2 or empty vector) per 6-well plate at a 1:2 DNA:reagent ratio, following the manufacturer’s protocol. Transfection efficiency (65.3 ± 4.1 %, mean ± SD) was validated by parallel GFP expression.

### 2.2 Quantitative PCR (qPCR) assays

RNA was reverse-transcribed by a type of M-MLV reverse transcriptase (M1701, Promega, USA). Quantitative PCR was performed by the use of an SYBR Ex Taq kit (638319, Takara, Japan). GAPDH: forward: 5′- TGT​GGG​CAT​CAA​TGG​ATT​TGG -3′, reverse: 5′- ACA​CCA​TGT​ATT​CCG​GGT​CAA​T -3’;SOX-2: 5′- CTC​GTG​CAG​TTC​TAC​TCG​TCG -3′, reverse: 5′- AGC​TCT​CGG​TCA​GGT​CCT​TT -3’; Nestin: 5′- CTG​CTA​CCC​TTG​AGA​CAC​CTG -3′, reverse: 5′- GGG​CTC​TGA​TCT​CTG​CAT​CTA​C -3’.

### 2.3 Immunoblotting

Cells were lysed using RIPA buffer (50 mM Tris-HCl pH 7.4, 150 mM NaCl, 1 % NP-40, 0.5 % sodium deoxycholate, 0.1 % SDS). Samples were separated by SDS-PAGE, and further transferred onto the PVDF membranes. The proteins were blocked with 5 % milk for 1 h. Primary antibodies including SRPX2 (1:1,000, ab91584, abcam), Bax (1:1,000, ab32503), Bcl-2 (1:1,000, ab182858), Caspase-3 (1:1,000, ab32351), cleaved-Caspase-3 (1:1,000, ab32402), Wnt3a (1:500, ab219412), beta-catenin (1:1,000, ab32572), Histone-H3 (1:1,000, ab32402), E-cadherin (1:1,000, ab32402), c-Myc (1:1,000, ab32402), Cyclin D1 (1:1,000, ab32402), and β-actin (1:3,000; ab8226), and then secondary antibodies were incubated for 1 h and photographed after the incubation with chemiluminescence.

### 2.4 Cell viability assay

3-(4,5-dimethylthiazol-2-yl)-2,5-diphenyltetrazolium bromide (MTT) assays were conducted to confirm the effects on cell viability. 1,000 cells/well NSCs were plated into 96-well plates and maintained for 48 h. Cells were plated into 96-well plates (1,000 cells/well) and cultured for 48 h. Treatments (transfection with plasmids and OGD/R conditions) were performed prior to cell viability assessment at the indicated time points. Cells were subsequently incubated with 3-(4,5-dimethylthiazol-2-yl)-2,5-diphenyltetrazolium bromide (MTT) for 4 h and dissolved with 200 uL DMSO. Then the OD490 value was measured.

### 2.5 Flow cytometry (FCM) assay

Cells were digested and resuspended in Annexin V and PI buffer for 5 min at room temperature away from light. Cell apoptosis were determined by a flow cytometer (BD, USA).

### 2.6 Superoxide dismutase (SOD), malondialdehyde (MDA), and MPO detection

After indicated treatment, cells were collected for detection of MDA, SOD, and MPO with relevant commercial kits (Jiancheng Bioengineering, China).

### 2.7 ROS staining and the Immunostaining of beta-catenin and SRPX2

The cells upon the indicated treatment were fixed and blocked with goat serum for 1 h. Slices were further incubated with DCF/ROS detection kit (ab238535, Abcam) or beta-catenin (1:200, ab32572), SRPX2 (1:1,000, ab91584, abcam), under the manufacture’s guidelines. After washing with PBS, the photos were captured. Fluorescence signals were captured under a fluorescence microscope. Although referred to as immunostaining previously, this assay is more accurately termed ROS fluorescence detection. Fluorescence intensity (Ex/Em = 485/535 nm) was quantified using ImageJ (v1.53) from ≥5 fields/group, normalized to cell count (DAPI). Raw values were expressed as fold-change versus untreated controls.

### 2.8 Immunofluorescence colocalization of SRPX2 with NSC markers

To assess the colocalization of SRPX2 with neural stem cell (NSC) markers, NSCs were plated onto poly-l-lysine-coated coverslips and treated under four conditions: Control + NC, Control + SRPX2, OGD/R + NC, and OGD/R + SRPX2. After treatment, cells were fixed with 4 % paraformaldehyde for 15 min and permeabilized with 0.1 % Triton X-100 in PBS for 10 min. Non-specific binding was blocked using 5 % goat serum for 1 h at room temperature. Cells were then incubated overnight at 4 °C with the following primary antibodies: anti-Nestin (1:200, Abcam, ab105389), anti-Sox2 (1:200, Abcam, ab97959), and anti-SRPX2 (1:500, Abcam, ab91584). After washing, cells were incubated with species-appropriate fluorescent-conjugated secondary antibodies (goat anti-rabbit Alexa Fluor 488, goat anti-mouse Alexa Fluor 594; both 1:1,000; Thermo Fisher Scientific) for 1 h at room temperature in the dark. Nuclei were counterstained with DAPI (1 μg/mL) for 5 min.

## 3 Statistics

All data were analyzed using GraphPad Prism 9.4.1. Normality was confirmed via Shapiro-Wilk tests (p > 0.05 for all groups unless noted), with homogeneity of variance verified by Levene’s test (p > 0.10). No outliers were excluded unless technical errors were documented during data collection. For non-normally distributed datasets, non-parametric Kruskal-Wallis with Dunn’s post hoc yielded concordant results. One-way ANOVA with Tukey’s post hoc (family-wise *α* = 0.05) was applied to normally distributed data. For all ANOVA analyses, data met assumptions of homogeneity of variance (Levene’s test p > 0.1) and near-normality (Shapiro-Wilk p > 0.05 unless noted). Where normality was borderline non-parametric Kruskal-Wallis with Dunn’s post-hoc confirmed ANOVA findings. Effect sizes (η^2^) are now reported alongside p-values.

## 4 Results

### 4.1 SRPX2 improves survival of OGD/R-stimulated NSCs

**Figure 1:**
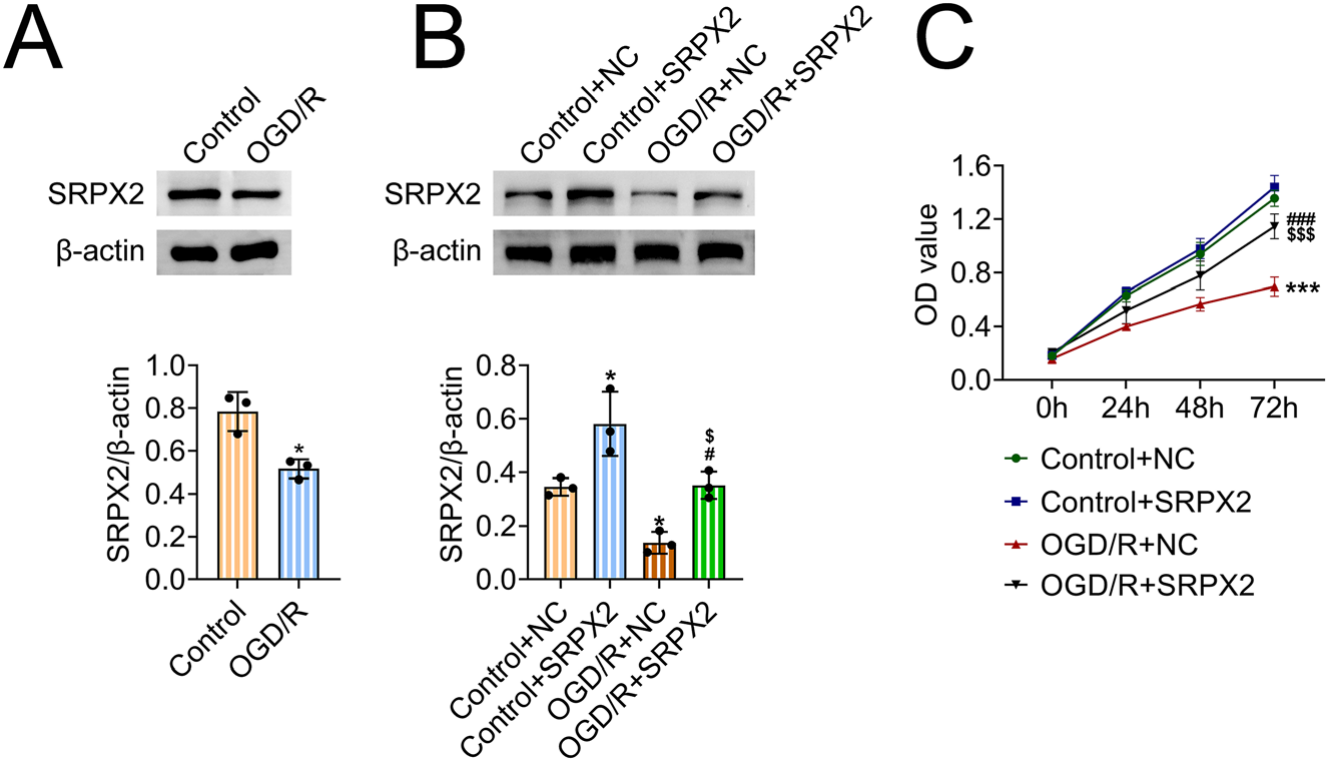
SRPX2 improves survival of OGD/R-stimulated NSCs. (A) Immunoblot exhibited the expression of SRPX2 in NSCs from control or OGD/R group. (B) Immunoblot exhibited the expression of SRPX2 in NSCs from the indicated groups. (C) MTT assays exhibited the survival of NSCs from the indicated groups. The OD490 value was measured. *p<0.05, ***p<0.001, OGD/R versus control, #*p*<0.05, ###*p*<0.001, OGD/R + SRPX2 versus control + SRPX2, $ *p*<0.05, $$$ *p*<0.001, OGD/R + SRPX2 versus OGD/R + NC. NC, negative control.

To uncover the possible role of SRPX2 in HIE progression, we first constructed a HIE cell model by the use of NSCs upon the treatment of OGD/R. Through Immunoblot assays, we noticed that SRPX2 was low expression in OGD/R-stimulated NSCs (Figure 1A). Subsequently, its overexpression plasmids were used to induce SRPX2 overexpression in control or OGD/R-stimulated NSCs. Immunoblot assays exhibited that the transfection of SRPX2 overexpression plasmids significantly increased its expression in control or OGD/R-stimulated NSCs (Figure 1B). Notably, performing MTT assays, we noticed OGD/R treatment suppressed the viability of NSCs (Figure 1C). However, SRPX2 overexpression promoted the viability of OGD/R-stimulated NSCs, with the increased OD450 value (Figure 1C). To further confirm the activity of SRPX2 in NSCs, immunofluorescence staining was conducted to assess the colocalization of SRPX2 with the NSC markers Nestin and Sox2 under normoxic and OGD/R conditions. As shown in Supplementary Figure S1A, Nestin was predominantly expressed in the cytoplasm of NSCs, and SRPX2 staining was also cytoplasmic. Merged images revealed SRPX2 co-localized with Nestin in both control and OGD/R-treated groups, with higher expression levels observed following SRPX2 overexpression. Similarly, Sox2 immunofluorescence revealed a nuclear localization pattern, while SRPX2 remained cytoplasmic (Figure S1B). Co-expression of Sox2 and SRPX2 in the same cells was clearly visible in merged images, indicating SRPX2 is present in NSCs and may exert biological activity within this population. Therefore, SRPX2 improves survival of OGD/R-stimulated NSCs.

### 4.2 SRPX2 inhibited apoptosis of OGD/R-stimulated NSCs

**Figure 2:**
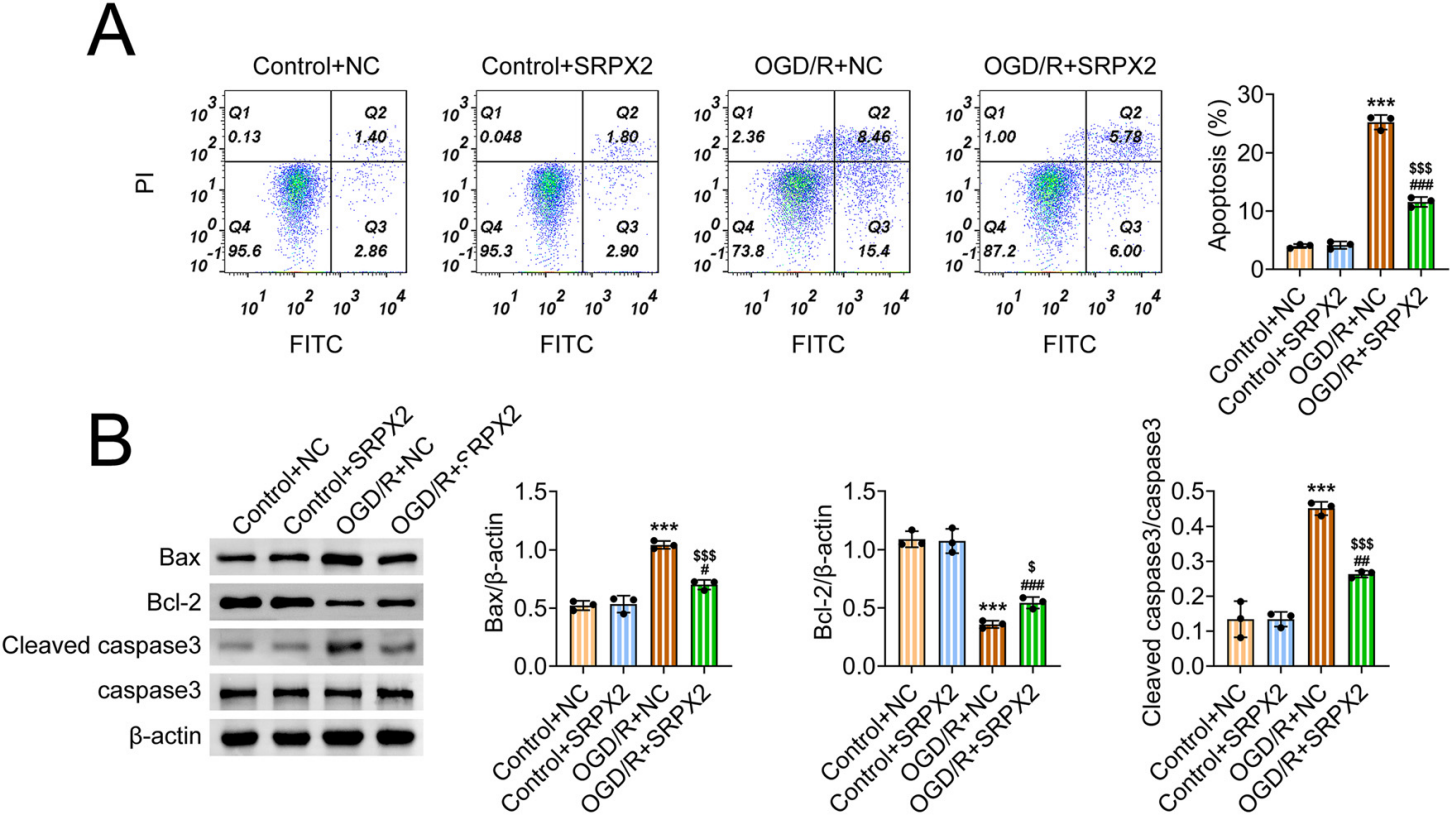
SRPX2 inhibited apoptosis of OGD/R-stimulated NSCs. (A) FCM assays exhibited the apoptosis levels of NSCs from the indicated groups. (B) Immunoblot exhibited the expression of Bax, Bcl-2, cleaved caspase-3, and caspase three in NSCs from control or OGD/R group. ***p<0.001, OGD/R versus control, ###*p*<0.001, OGD/R + SRPX2 versus control + SRPX2, $ *p*<0.05, $$$ *p*<0.001, OGD/R + SRPX2 versus OGD/R + NC. NC, negative control.

Subsequently, the effects of SRPX2 on the apoptosis of NSCs were further investigated. Performing FCM assays, we noticed OGD/R stimulated the apoptosis of NSCs (Figure 2A). SRPX2 overexpression had modest effects on the apoptosis of NSCs, however, its overexpression significantly suppressed the apoptosis of OGD/R-stimulated NSCs (Figure 2A). Immunoblot assays exhibited that OGD/R treatment increased the expression of Bax and cleaved caspase-3, and decreased Bcl-2 expression in NSCs (Figure 2B). Whereas SRPX2 overexpression suppressed the expression of Bax and cleaved caspase-3, and increased Bcl-2 expression in OGD/R-stimulated NSCs (Figure 2B). Therefore, SRPX2 inhibited apoptosis of OGD/R-stimulated NSCs.

### 4.3 SRPX2 restrained oxidative stress of OGD/R-stimulated NSCs

**Figure 3:**
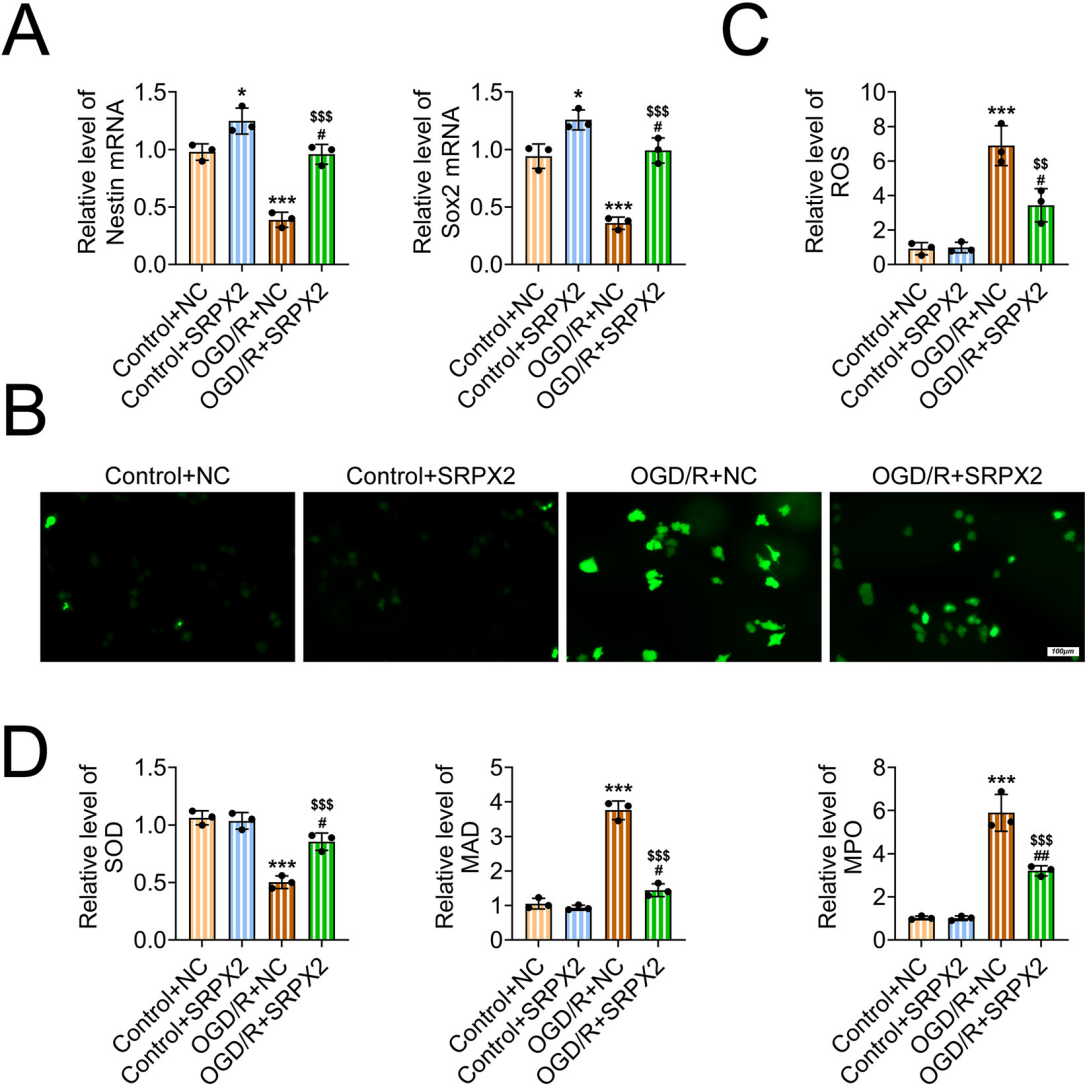
SRPX2 restrained oxidative stress of OGD/R-stimulated NSCs. (A) qPCR assays showed the mRNA levels of Nestin and SOX-2 in NSCs from the indicated groups with or without OGD/R treatment. (B, C) immunostaining exhibited the ROS levels in NSCs from the indicated groups with or without OGD/R treatment (B). (C). The quantification of ROS intensity was conducted. (D) ELISA exhibited the secretion of SOD, MDA, and MPO in NSCs from the indicated groups with or without OGD/R treatment. ***p<0.001, OGD/R versus control, #*p*<0.05, ##*p*<0.01, OGD/R + SRPX2 versus control + SRPX2, $$ *p*<0.01, $$$ *p*<0.001, OGD/R + SRPX2 versus OGD/R + NC. NC, negative control.

We detected the effects of SRPX2 on the oxidative stress of OGD/R-stimulated NSCs. First, we detected the mRNA levels of the markers of NSCs, including Nestin and SOX-2 in the indicated groups via qPCR assays. OGD/R treatment decreased the mRNA levels of these markers, whereas SRPX2 increased the mRNA levels of these markers (Figure 3A). We further noticed the increased ROS levels in OGD/R-stimulated NSCs, with the strongly staining of ROS (Figure 3B and C). However, SRPX2 overexpression suppressed the ROS levels in OGD/R-stimulated NSCs (Figure 3B and C). We noticed that OGD/R decreased the secretion of SOD and increased the secretion of MAD and MPO, suggesting the promotion of oxidative stress (Figure 3D). However, overexpression of SRPX2 increased the secretion of SOD and decreased the secretion of MAD and MPO in OGD/R-stimulated NSCs (Figure 3B). Therefore, SRPX2 restrained oxidative stress of OGD/R-stimulated NSCs.

### 4.4 SRPX2 activated the wnt/beta-catenin pathway

**Figure 4:**
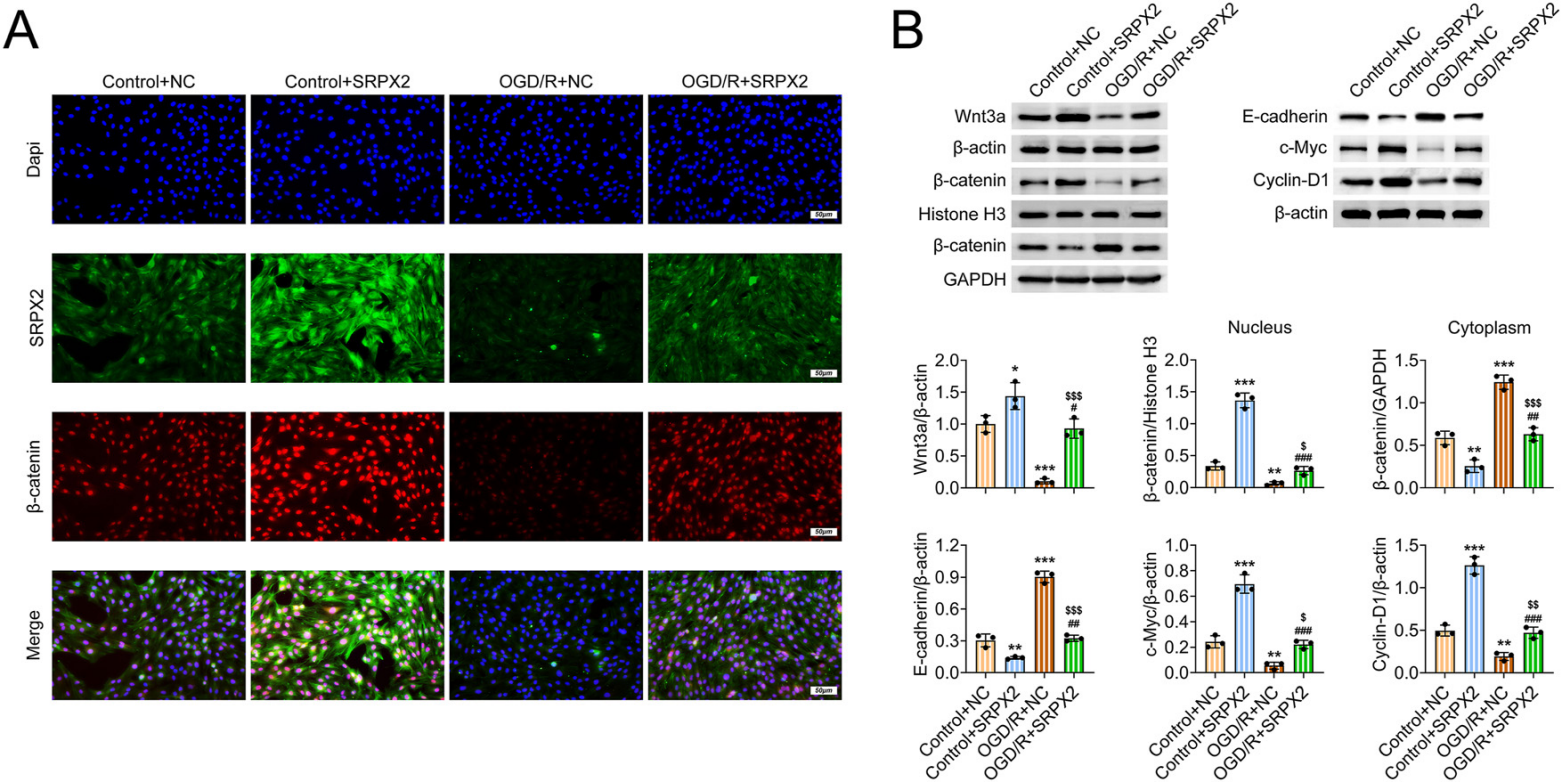
SRPX2 activated the wnt/beta-catenin pathway. (A) Immunostaining exhibited the expression of SRPX2 and beta-catenin of the NSCs from the indicated groups. Scale bar, 50 μm. (B) Immunoblot exhibited the expression of wnt3a, beta-catenin, E-cadherin, c-Myc, and Cyclin D1 in NSCs or the nucleus of the NSCs from the indicated groups. **p<0.01, ***p<0.001, OGD/R versus control, #*p*<0.05, ##*p*<0.01, OGD/R + SRPX2 versus control + SRPX2, $ *p*<0.05, $$ *p*<0.01, $$$ *p*<0.001, OGD/R + SRPX2 versus OGD/R + NC. NC, negative control.

Then the possible mechanism was explored through Immunostaining and Immunoblot assays. Immunostaining assays showed that OGD/R treatment decreased the expression of SRPX2 in NSCs, whereas overexpression of SRPX2 increased its expression (Figure 4A). Interestingly, OGD/R treatment also decreased the expression of beta-catenin, and SRPX2 overexpression increased the expression of beta – catenin (Figure 4A).

Immunoblot showed that OGD/R treatment decreased the expression of Wnt-3α and beta – catenin in NSCs, whereas SRPX2 overexpression increased the expression of Wnt-3α and beta – catenin (Figure 4B). However, OGD/R treatment increased the nucleus localization of beta – catenin, whereas SRPX2 overexpression decreased its nucleus localization (Figure 4B). Furthermore, the expression of downstream proteins, including c-Myc and Cyclin D1, was decreased and E-cadherin was increased caused by OGD/R, whereas SRPX2 overexpression reversed the alterations of the expression of these proteins (Figure 4B). Therefore, SRPX2 activated the Wnt/beta-catenin pathway in OGD/R-stimulated NSCs.

## 5 Discussion

HIE is a brain lesion caused by various causes of brain tissue ischemia and hypoxia, the most common is neonatal hypoxic encephalopathy [1], 17]. So far, there is no complete and unified treatment plan for HIE [18]. Hypoxia ischemia can cause two different types of cell death, namely necrosis and apoptosis. Delayed neuronal death is essentially apoptosis, and the expression of a series of apoptosis-related genes has been detected in animal models [19], 20]. During hypoxia and ischemia, a large number of oxygen free radicals accumulate in the body, damaging cell membranes, proteins and nucleic acids, resulting in the destruction of cell structure and function [21]. The pathogenesis of HIE is very complex and is the result of a series of biochemical chain reactions caused by the comprehensive action of various mechanisms [22]. Herein, NSC cell OGD/R model was established as a HIE cell model. We revealed that SRPX2 can suppress neuronal apoptosis and oxidative stress, and alleviate neuronal damage.

With the progress and development of medicine, stem cell therapy has brought more new hopes for the treatment of HIE, and is of great significance in neuroprotection and restoring the function of brain damaged cells and tissues [5]. In recent years, the regeneration of NSCs in nerve repair and tissue injury has been widely used in the treatment of nervous system diseases, including ischemic stroke and craniocerebral injury, with obvious efficacy [23]. Exogenous NSCs can promote neuronal regeneration and reduce inflammatory response, which brings light to the treatment of HIE [24]. Herein, the NSCs have been isolated from hippocampus tissues of Sprague-Dawley (SD) rat. We therefore noticed the role of SRPX2 in NSCs.

SRPX2 is a kind of chondroitin sulfate proteosaccharide, which is vital in the development of brain language center [25]. SRPX2 is involved in many biological behaviors such as seizures, angiogenesis, and cell adhesion [12]. It is normally expressed in brain neurons, heart, lungs, trachea and other normal tissues, the mutation of this gene will lead to seizures, speech loss, etc., affecting the development of the normal nervous system [25] Subsequent studies have found that SRPX2 can promote angiogenesis and cell adhesion [13]. Our data confirmed that SRPX2 can improve neuron apoptosis and oxidative stress, and alleviate neuronal injury.

SRPX2 demonstrated protective effects against apoptosis and oxidative stress in NSCs similar to established neuroprotective drugs, such as edaravone, melatonin, and resveratrol. Edaravone exerts its neuroprotective effects primarily through scavenging reactive oxygen species (ROS), while melatonin and resveratrol modulate multiple pathways, including antioxidant, anti-apoptotic, and anti-inflammatory mechanisms [13]. Although SRPX2 shares antioxidative and anti-apoptotic properties with these drugs, it uniquely targets the Wnt/β-Catenin pathway, highlighting a distinct neuroprotective mechanism. Future comparative studies would be beneficial to precisely position SRPX2 relative to these established therapeutic agents.

SRPX2’s neuroprotective effects are quantitatively similar to those reported for clinically relevant agents like edaravone and melatonin in mitigating apoptosis and oxidative stress in hypoxic-ischemic models [26]. While these compounds act through distinct mechanisms (e.g., ROS scavenging or receptor-mediated signaling), SRPX2’s unique activation of the Wnt/β-catenin pathway offers a complementary therapeutic strategy [27], 28]. Further comparative studies will help clarify its relative potency and potential synergies with existing treatments.

Similar to other extracellular matrix-associated proteins reported to influence neural stem cell function, such as CSPGs, SRPX2 appears to exert protective effects via modulation of signaling pathways critical for cell survival under ischemic conditions [25], 29]. These parallels suggest SRPX2’s potential role as part of a broader class of molecules that support neural survival and regeneration following ischemic injury.

Oxidative stress is an important pathological reaction process of ischemic cerebrovascular diseases [30]. After ischemic brain injury, ROS increase, which causes cell death through necrosis or apoptosis, and indirectly leads to cell apoptosis by mediating mitochondrial diameter and nuclear transcription factor signal transduction [31]. ROS produced by oxidative stress can also regulate the activity of transcription factors and is vital in the signal transduction of apoptosis [31]. Through Immunostaining and ELISA, we revealed that SRPX2 inhibits oxidative stress of OGD/R-stimulated neurons. We hypothesize that SPRX2 inhibits apoptosis and neuronal damage by inhibiting oxidative stress.

Wnt signaling molecules are vital in regulating the proliferation, differentiation and fate determination of stem cells, including NSCs [32]. β-catenin is a key molecule in the Wnt signaling pathway, and plays a key role in regulating the growth, differentiation and displacement of NSCs in ischemic brain injury [33]. Our data exhibited that SRPX2 activates the Wnt/beta-catenin axis in OGD/R-stimulated neurons, suggesting that Wnt/beta-catenin axis is critical in HIE progression. Wnt3a was selected because it is a classical ligand known to activate canonical Wnt signaling, especially within neural stem cell contexts. While our findings emphasize the Wnt/β-catenin pathway, the potential involvement of SRPX2 in other neuroprotective pathways, such as PI3K/Akt, NF-κB, and MAPK signaling, cannot be ruled out and deserves further exploration. Future studies should investigate the interaction between SRPX2 and these pathways to fully elucidate its neuroprotective mechanisms. The exact mechanism by which SRPX2 activates the Wnt/β-Catenin pathway remains unclear, specifically whether SRPX2 interacts directly with components of this pathway or functions via intermediate signaling molecules. Identifying these intermediate interactions should be an essential direction for future research.

Our study demonstrates that SRPX2 overexpression is associated with reduced apoptosis and oxidative stress in OGD/R-injured NSCs, correlating with modulation of Wnt/β-catenin signaling. While these findings suggest SRPX2’s potential neuroprotective role, further research is needed to fully elucidate the underlying molecular mechanisms, including whether SRPX2 directly interacts with Wnt pathway components or influences β-catenin dynamics through indirect regulation.

While we focused on Wnt/β-catenin due to its pronounced dysregulation in our model, we acknowledge that SRPX2 may engage alternative pathways (e.g., PI3K/Akt) in other cell types or injury contexts [34], 35]. This selectivity mirrors recent findings that chondroitin sulfate proteoglycans exhibit pathway specificity depending on cellular microenvironment. Notably, Wnt/β-catenin activation can crosstalk with PI3K/Akt and NF-κB (via GSK3β inhibition), potentially explaining our observed anti-apoptotic effects without excluding broader pathway involvement [36]. Future phosphoproteomic studies will systematically map SRPX2’s signaling network.

While our data demonstrate SRPX2-mediated activation of Wnt/β-catenin signaling, the precise molecular interaction – whether through direct binding to Wnt ligands/receptors or via intermediate adaptors – remains to be resolved. This mechanistic ambiguity represents a key limitation of the current study. Notably, prior studies implicate chondroitin sulfate proteoglycans in modulating Wnt pathway activity through extracellular matrix remodeling [37], 38]. Given SRPX2’s structural homology to CSPGs, it may similarly orchestrate Wnt signaling via ECM-dependent scaffolding, a hypothesis warranting further investigation.

While our current findings highlight Wnt/β-catenin as a primary pathway, SRPX2’s structural domains share homology with proteins known to regulate PI3K/Akt and NF-κB. This raises the intriguing possibility that SRPX2 may coordinate multiple neuroprotective signals, akin to pleiotropic factors like BDNF, though empirical validation is needed.

Due to technical and resource constraints, performing animal experiments at this stage is beyond the scope of the current manuscript. However, we agree that future research incorporating animal models would be valuable to fully validate our findings. In addition, while our findings using the NSC-based OGD/R model suggest that SRPX2 plays a neuroprotective role, assessing SRPX2 expression levels in clinical brain tissues across varying degrees of injury severity remains important. While our *in vitro* OGD/R model provides mechanistic insights, the therapeutic potential of SRPX2 must ultimately be validated *in vivo*. Future studies employing established HIE models will be critical to assess SRPX2’s ability to mitigate brain injury and improve functional outcomes. Given SRPX2’s demonstrated neuroprotective effects in HIE, its potential application in other CNS disorders characterized by oxidative stress and apoptosis – such as ischemic stroke or Alzheimer’s disease – warrants investigation. Notably, the Wnt/β-catenin pathway, which we identified as a key mediator of SRPX2’s effects, is also implicated in blood-brain barrier repair post-stroke and amyloid clearance, suggesting plausible mechanistic overlap.

In summary, SRPX2 suppressed OGD/R-stimulated neuronal apoptosis and oxidative stress, and alleviated neuronal injury.

## Supplementary Material

Supplementary Material

Supplementary Material
