# Supplementary material for: SRPX2 exhibits neuroprotective effects in neural stem cells: inhibition of OGD/R-stimulated apoptosis and oxidative stress
Source: Open Life Sci. 2026 Jan 13;21(1):20251182. doi: 10.1515/biol-2025-1182 (PMC12917582; doi:10.1515/biol-2025-1182)
Supplement: Supplementary file 2 — Supplementary Material [file j_biol-2025-1182_suppl_002.docx]

Figure S1. SRPX2 colocalizes with NSC markers Nestin and Sox2 in OGD/R-injured neural stem cells. (A) Immunofluorescence staining of NSC marker Nestin (red), SRPX2 (green), and DAPI (blue) in NSCs from four groups: Control+NC, Control+SRPX2, OGD/R+NC, and OGD/R+SRPX2. (B) Immunofluorescence staining of Sox2 (red), SRPX2 (green), and DAPI (blue) in NSCs from the same groups. SRPX2 expression was primarily cytoplasmic, while Nestin was cytoplasmic and Sox2 nuclear. Merged images demonstrate SRPX2 co-localization with NSC markers. Scale bar = 50 µm.
